# Maternal and perinatal outcomes among women with hypertensive disorders in pregnancy in Kumasi, Ghana

**DOI:** 10.1371/journal.pone.0223478

**Published:** 2019-10-04

**Authors:** Edward T. Dassah, Eunice Kusi-Mensah, Emmanuel S. K. Morhe, Alexander T. Odoi

**Affiliations:** 1 School of Public Health, Kwame Nkrumah University of Science and Technology, Kumasi, Ghana; 2 Department of Obstetrics and Gynaecology, Komfo Anokye Teaching Hospital, Kumasi, Ghana; 3 Transfusion Medicine Unit, Komfo Anokye Teaching Hospital, Kumasi, Ghana; 4 School of Medicine, University of Health and Allied Sciences, Ho, Ghana; University of Ghana, GHANA

## Abstract

**Background:**

Data pertaining to maternal and perinatal outcomes associated with the complete spectrum of hypertensive disorders in pregnancy (HDPs) is sparse in low resource settings. This study aimed to determine adverse maternal and perinatal outcomes among women admitted with HDPs in a tertiary hospital in Ghana, and directly compare these outcomes among women with pre-eclampsia/eclampsia and those with chronic/gestational hypertension.

**Methods:**

An analytical cross-sectional study was conducted among women who were admitted with HDPs to Komfo Anokye Teaching Hospital from July 1, 2014 to September 30, 2014. Data was collected on their socio-demographic and reproductive characteristics using a pretested structured questionnaire and review of their antenatal records. Crude and adjusted relative risks (RRs), with 95% confidence intervals (CIs), associated with adverse maternal and perinatal outcomes were compared using multivariable binomial regression. P ≤0.05 was considered statistically significant.

**Results:**

A total of 451 women with HDPs were studied: 5.3%, 32.4%, 48.8% and 13.5% had chronic hypertension, gestational hypertension, pre-eclampsia and eclampsia respectively. Over 80% were either referrals or “self-referred” from other facilities. Overall, 87% had adverse maternal or perinatal outcomes. Women with pre-eclampsia/eclampsia were at increased risks of caesarean section (adjusted RR, 1.37; 95% CI, 1.01–1.87), preterm delivery at <34 weeks’ gestation (adjusted RR, 2.74; 95% CI, 1.40–5.36) and preterm delivery at <37 weeks’ gestation (adjusted RR, 1.89; 95% CI, 1.25–2.85), compared to women with chronic/gestational hypertension.

**Conclusion:**

Women with pre-eclampsia/eclampsia were at higher risk of adverse pregnancy outcome compared to those with chronic/gestational hypertension. Strategies for prevention and management of pre-eclampsia/eclampsia to improve pregnancy outcomes are required in this major maternity care centre.

## Introduction

Hypertensive disorders in pregnancy (HDPs) comprising chronic hypertension, gestational hypertension, pre-eclampsia and eclampsia complicate 4–10% of pregnancies worldwide[1]. They account for about 15% of all maternal deaths globally, almost all (>99%) of which occur in developing countries[1]. About 25% of perinatal deaths in these settings are attributable to HDPs[2]. These disorders have been reported as a leading cause of maternal mortality in sub-Saharan Africa, including most tertiary and rural hospitals in Nigeria[3] and major tertiary health facilities in Ghana as well as other countries[4, 5]. In particular, they have remained as the main cause of maternal deaths in Komfo Anokye Teaching Hospital (KATH) for almost two decades[6]. Although the principles of managing HDPs are the same the world over, the disproportionately high adverse pregnancy outcomes in resource-constrained settings are mainly due to challenges associated with the management and quality of care of HDPs in these settings[1, 7]. Even within countries, especially so in sub-Saharan Africa, differences exist in pregnancy outcomes due to socio-cultural differences and variations in the distribution and quality of health care[1].

A number of studies have been conducted in well-resourced settings to evaluate maternal and perinatal outcomes associated with the complete spectrum of HDPs[8–10]. However, such data is sparse in low resource settings including sub-Saharan Africa, where most studies have focused on pre-eclampsia or eclampsia possibly because of increased risks of adverse outcomes. The few published studies on pregnancy outcomes associated with the complete spectrum of HDPs in sub-Saharan Africa have focused on either maternal[5] or perinatal[11] outcomes and may be further limited by the lack of risk estimates (thereby failing to adjust for confounding factors) for adverse outcomes associated with specific HDPs[11]. Also, the interventions, resources and expertise required to manage women with chronic/gestational hypertension and those with pre-eclampsia/eclampsia could be quite different and may not be readily available in all settings. Therefore, this study aimed to determine adverse maternal and perinatal outcomes among women admitted with HDPs in a tertiary hospital in Ghana, and directly compare these outcomes among women with pre-eclampsia/eclampsia and those with chronic/gestational hypertension in this low resource setting.

## Methods

This was an analytical cross-sectional study conducted among women with HDPs admitted to the high dependency unit of the department of Obstetrics and Gynaecology, KATH from July 1, 2014 to September 30, 2014. The high dependency unit is dedicated to the management of HDPs and similar conditions. As such, all cases of HDPs which require admission or delivery, are admitted to the unit. All women with an estimated gestational age (EGA) of at least 20 weeks who were admitted to the unit within the study period were eligible for inclusion in the study. Women without antenatal records were excluded since important data including results of investigations pertaining to the antenatal period usually contained in the antenatal record were not available.

KATH is the second largest hospital in Ghana, and the main tertiary and referral health facility in the northern sector of the country, serving five out of the 10 regions of the country[4]. It is located in Kumasi, the regional capital of Ashanti. The Obstetrics and Gynaecology department of the hospital has a bed capacity of 160, conducts about 10,000 deliveries a year and admits an average of 150 patients with HDPs monthly to its High Dependency Unit[12].

### Definitions

Hypertension in pregnancy was defined as a systolic blood pressure of at least 140 mmHg or a diastolic blood pressure of at least 90 mmHg[7, 13]. Hypertension was considered severe if the SBP ≥160 mmHg or DBP ≥110 mmHg. Blood pressure measurements were taken on more than one occasion at least 4 hours apart, with the patient sitting up or in the left lateral recumbent position with her arm at the level of her heart using a mercury sphygmomanometer with an appropriate-sized cuff. Using semi-quantitative urine dipsticks, proteinuria of at least 1+ in the presence of hypertension without evidence of urinary tract infection was considered significant[7, 13]. HDPs were classified as: gestational hypertension, chronic hypertension, pre-eclampsia superimposed on chronic hypertension, pre-eclampsia or eclampsia[7]. Chronic hypertension in pregnancy was defined as hypertension predating the pregnancy or diagnosed before the 20^th^ week of pregnancy. Gestational hypertension was hypertension without proteinuria detected for the first time after 20 weeks of pregnancy. Women were considered to have pre-eclampsia if they had hypertension with significant proteinuria (≥1+ reading on urine dipstick) occurring after 20 weeks of gestation. Pre-eclampsia was considered severe if at least one of the following criteria were present; hypertension with severe proteinuria (2+, 3+ or 4+ on urine dipstick) or evidence of multiorgan impairment. Pre-eclampsia superimposed on chronic hypertension referred to worsening of the hypertension, new-onset proteinuria or development of organ system impairment in women with hypertension before 20 weeks of gestation[7, 13]. Women with pre-eclampsia who had seizures that could not be attributed to other causes were considered to have eclampsia[7]. For women’s whose condition progressed from one stage to the other (e.g. from chronic hypertension to eclampsia), the latter was taken as the final diagnosis. The final diagnosis of the HDP was determined at KATH.

Stillbirth was defined as death of a fetus weighing at least 1000g or 28 completed weeks of gestation occurring at the complete expulsion or extraction from its mother in accordance with the World Health Organization’s agreed definition of stillbirth for international comparison[14]. Any pregnancy that terminated before 28 completed weeks was considered as an abortion. Perinatal deaths were stillbirths and newborn deaths within the first seven days of delivery. Preterm deliveries were births of infants occurring after 28 completed weeks but before 37 completed weeks of gestation[11]. A low Apgar score was any score less than 7[11]. Prolonged maternal hospital stay was defined as more than three days for vaginal births and more than four days for caesarean deliveries[8]. EGA was the number days between the first day of the last menstrual period and the date of delivery expressed in completed weeks using ultrasound or clinical assessment as documented in the antenatal record.

### Management of HDPs at KATH

Once the diagnosis of HDP was established, the key principles of management were; management of hypertension, management and prevention of complications including eclamptic fits and safe delivery with good outcome for mother and baby[7]. Antihypertensive therapy was initiated at blood pressure values of 150/100 mmHg or higher. The commonly used antihypertensives were nifedipine and methyldopa. Second line antihypertensives were labetalol and hydralazine. Parenteral hydralazine was the drug of choice for severe acute hypertension followed by parenteral labetalol. For women with severe pre-eclampsia or eclampsia, intermittent intramuscular magnesium sulphate (Pritchard regimen) was given for the prevention and management of eclamptic fits respectively. This comprised a loading dose of 14 g (given as 4 g intravenously over 5–10 minutes plus 10 g intramuscularly, 5 g into each buttock) and a maintenance dose of 5 g intramuscularly into alternate buttocks every 4 hours till 24 hours after delivery or the last fit (which ever came last). Women with severe HDPs between 28 and 34 weeks were given a maximum of two courses of intramuscular dexamethasone (one week apart) to promote fetal lung maturity. The timing and mode of delivery were determined by the maternal and fetal conditions.

### Study procedures

Signed informed consent was obtained from eligible participants; witnessed consent was offered when a participant was not able to read the information sheet and thumbprints accepted when necessary. In the case of minors (girls under 18 years of age), informed consent and assent were obtained from the parent/guardian and the patient respectively. Consent was sought from the spouse/parents of unconscious patients.

All consenting women had their antenatal cards/maternal record booklets reviewed and underwent a confidential interview (where possible) in vernacular or English using a pretested structured questionnaire. Information was collected on their socio-demographic characteristics and the care received during pregnancy and delivery. For unconscious patients, supplementary history was obtained from spouses/parents where necessary. Women who had not delivered were followed up at delivery. All recruited clients and their newborns had their records reviewed by a member of the research team for maternal and neonatal morbidity/mortality on their first postnatal visit, which was usually within one week of being discharged. Newborns who were admitted at the neonatal intensive care unit (NICU) also had their records reviewed within one week of delivery. Pretesting of the data collection tools was carried out among women admitted for HDPs at the regional hospital, prior to the actual data collection.

#### Sample size calculations and statistical analysis

Sample size calculations were done in Epi Info^TM^ version 7.1.1.14 (Centers for Disease Control and Prevention (CDC); USA) at 80% power and 95% confidence interval. Assuming the proportions of the various HDPs were similar to those observed by Crenstil[15], chronic/gestational hypertension (23.3%) and pre-eclampsia/eclampsia (76.7%), an estimated sample size of 437 was adequate to detect an absolute difference of 15% in adverse maternal or adverse fetal outcomes between women with pre-eclampsia/eclampsia and those with chronic/gestational hypertension. Making allowance for 5% contingency, our estimated sample size was 459.

Data was analysed in Stata 11.0 (Stata Corporation, Texas, USA). Categorical variables were compared using the Chi-square (χ^2^) or Fisher’s exact tests as appropriate, while continuous variables were compared using student t-tests. Risk factors associated with adverse maternal and perinatal outcomes were examined using binomial regression with a log-link function to estimate crude and adjusted relative risks (RRs) with 95% confidence intervals (CIs). Variables for the regression models were selected on the basis of biologic plausibility, evidence from the literature and results of the univariable analysis. Potential interaction effects of relevant covariates and statistical significance were assessed using the likelihood ratio and Wald tests. To directly compare outcomes in women with pre-eclampsia/eclampsia with those of women with chronic/gestational hypertension, crude and adjusted RRs were calculated for pre-eclampsia/eclampsia relative to chronic/gestational hypertension. All missing values were excluded from the analyses. Tests with probability values ≤0.05 were considered statistically significant.

### Ethical approval

The study was approved by the Committee on Human Research, Publications and Ethics of the School of Medical Sciences, Kwame Nkrumah University of Science and Technology, and KATH, Kumasi (Protocol number CHRPE/AP/160/13, approved on 05/07/2013). Written informed consent was also obtained from each study participant.

## Results

A total of 474 women with HDPs were admitted to the High Dependency Unit of the department within the study period, of whom 453 were recruited into the study. Twenty-one women (4.4%) were excluded for the following reasons; 7 were discharged and had left the ward before visits by the research team, 6 were admitted and discharged before 20 weeks of gestation, and 8 did not have their antenatal records on admission or were non-attendees. Two women who enrolled into the study and discharged prior to delivery were lost to follow-up, leaving data on 451 women for analysis.

### Socio-demographic and reproductive characteristics

The socio-demographic and reproductive characteristics of the women are compared in Table 1. The socio-demographic characteristics of women with pre-eclampsia/eclampsia were not statistically significantly different from those with chronic/gestational hypertension. However, with respect to their reproductive characteristics, women with pre-eclampsia/eclampsia were significantly more likely to be carrying their first pregnancy with their index partner, referred or admitted to KATH antepartum or postpartum, to have had most ANC in health care facilities other than KATH, and received less than 4 antenatal visits prior to admission at KATH, compared to those with chronic/gestational hypertension. Conversely, women with chronic/gestational hypertension were significantly more likely to have had most ANC and be admitted during labour at KATH, compared to those with pre-eclampsia/eclampsia.

**Table 1:** Comparison of socio-demographic and reproductive characteristics among women with chronic/gestational hypertension and those with pre-eclampsia/eclampsia.

Variable	Chronic/Gestational Hypertension (n = 170) n (%)^a^	Pre-eclampsia/ Eclampsia (n = 281) n (%)^a^	P-value
Age group (completed years)			0.14
15–19	4 (4.7)	24 (8.5)	
20–34	120 (70.6)	204 (72.6)	
35–49	42 (24.7)	53 (18.9)	
Mean (standard deviation)	30.3 (6.16)	28.1 (6.38)	
Educational background			0.70
No formal education	10 (5.8)	14 (5.0)	
Basic education	104 (61.2)	164 (58.4)	
Post basic education	56 (32.9)	103 (36.7)	
Marital status			0.12
Single	7 (4.2)	22 (7.8)	
Married/cohabiting	168 (95.9)	259 (92.2)	
Religion			0.22
Christianity	127 (74.4)	224 (79.7)	
Islam	43 (25.3)	57 (20.3)	
Occupation			0.44
Unemployed	23 (13.6)	51 (18.2)	
Semi-skilled	117 (69.2)	187 (66.5)	
Skilled	29 (17.2)	43 (15.3)	
Primipaternity			0.01
Yes	50 (29.6)	118 (42.5)	
No	119 (70.4)	160 (57.5)	
Parity			0.10
0	51 (30.0)	108 (38.4)	
1–4	104 (61.2)	158 (56.2)	
5–8	15 (8.8)	15 (5.3)	
Type of gestation			0.73
Singleton (1)	157 (92.4)	257 (91.5)	
Multifetal (2 or 3)	13 (7.7)	24 (8.5)	
EGA at booking (completed weeks)			0.11
≤13 (first trimester)	96 (56.5)	138 (49.1)	
14–26 (second trimester)	65 (38.2)	134 (47.7)	
≥27 (third trimester)	9 (5.3)	9 (3.2)	
Mean (standard deviation)	14^+0^ (6^+4^)	14^+4^ (6^+1^)	
EGA at diagnosis (completed weeks)			0.07
<26	34 (20.9)	35 (14.0)	
≥27	129 (79.1)	215 (86.0)	
Mean (standard deviation)	32^+6^ (10^+1^)	34^+0^ (7^+0^)	
Type of facility most ANC was received			<0.001
Private health facility	41 (24.1)	99 (35.2)	
Other public health facility	82 (48.2)	154 (54.8)	
KATH	47 (27.7)	28 (10.0)	
Number of antenatal visits before admission			0.01
<4	12 (7.1)	44 (16.1)	
≥4	156 (92.9)	230 (83.9)	
Median (interquartile range)	8 (6–10)	6 (4–8)	
Stage of pregnancy at admission			0.01
Antepartum	81 (47.6)	159 (56.6)	
Intrapartum	78 (45.9)	89 (31.7)	
Postpartum	11 (6.5)	33 (11.7)	

^a^Values are given as number (percentage) unless otherwise indicated.

EGA, estimated gestational age; ANC, antenatal care; KATH, Komfo Anokye Teaching Hospital.

The mean EGA at booking was 14 weeks 3 days; only 4% of the women booked in the third trimester. Less than one-fifth (16.6%) of the women received ANC at KATH, 69.9% were referred, and 13.5% “self-referred” themselves to KATH. Over 80% of the women were diagnosed with HDPs in the third trimester (mean EGA at diagnosis 33weeks 4 days). The mean EGA at delivery and birth weight were 37weeks 2 days (standard deviation, 26 days) and 2.6kg (standard deviation, 0.84kg). There were more newborn males than females at birth (52.4% vs. 47.6%).

Laboratory investigations were not done for some patients on admission commonly due to stockouts of reagents. For example, haematological tests were not done for 98 (21.7%) women. Altogether, 24 (5.3%), 146 (32.4%), 220 (48.8%) and 61 (13.5%) women had chronic hypertension, gestational hypertension, pre-eclampsia and eclampsia respectively. Sixteen women with pre-eclampsia had chronic hypertension with superimposed pre-eclampsia, and 6 women with eclampsia developed the condition after delivery.

Table 2 summarises the adverse maternal and perinatal outcomes of the women according to HDP categories. Pregnancy outcomes of second twins and second/third triplets in cases of multifetal gestations were excluded. There were 35 twin and 2 triplet deliveries. Except for low Apgar score at one 1-minute, low birth weight of 1.0–2.4 kg and stillbirths, women with gestational hypertension had the least proportions of adverse outcomes. These proportions were generally highest in the eclampsia or chronic hypertension groups; women with pre-eclampsia had the highest proportion of extreme preterm births and stillbirths. About half (51.3%) of the women had caesarean sections and 37.7% had prolonged maternal stay at KATH. About 3% of the pregnancy outcomes were abortions and nearly a third (30.4%) were preterm births. Only 2 of the abortions were alive at the time of expulsion; both were admitted to NICU but died within 12 hours. The mean EGA at delivery was 37 weeks 2 days (standard deviation, 3 weeks 5 days). About 38% of live births had low Apgar scores of <7 in the first minute. The prevalence of low Apgar scores improved significantly to 9.7% after 5 minutes (37.8% vs. 9.7%; p<0.001). Nearly 4 out of 10 newborns (37.6%) were low birth weight. The mean birth weight was 2.6 kg (standard deviation, 0.84 kg). About one quarter (26.2%) of the newborns were admitted to NICU. Altogether, there were 84 (18.8%) abortions/perinatal deaths with 51 (11.4%) being stillbirths (in-utero fetal demise after 28 completed weeks). Two-thirds (34) of the stillbirths occurred prior to onset of labour and the remaining third (17) died during labour and delivery. Overall, 87% of the women had adverse maternal or fetal outcomes.

**Table 2:** Adverse maternal and fetal outcomes.

	Type of hypertensive disorder				
Pregnancy outcomes	Chronic hypertension (n = 24) n (%)	Gestational hypertension (n = 146) n (%)	Pre-eclampsia (n = 220) n (%)	Eclampsia (n = 61) n (%)	Total (N = 451) n (%)^a^
*Maternal outcomes*					
Caesarean section	17 (70.8)	55 (37.7)	122 (55.5)	37 (61.7)	231(51.3)
Prolonged maternal hospital stay	12 (50.0)	34 (23.3)	95 (43.2)	29 (47.5)	170 (37.7)
Maternal mortality	0 (0.0)	0 (0.0)	4 (1.8)	6 (9.8)	10 (2.2)
*Fetal outcomes*					
EGA at abortion/delivery (completed weeks)^b^					
21–27	1 (4.2)	0 (0.0)	8 (3.7)	4 (7.4)	13 (2.9)
28–33	3 (12.5)	7 (4.8)	39 (17.8)	7 (13.0)	56 (12.7)
34–36	5 (20.8)	20 (13.8)	38 (17.4)	15 (27.8)	78 (17.7)
37–43	15 (62.5)	118 (81.4)	134 (61.2)	28 (51.9)	295 (66.7)
Low Apgar score (<7) at 1 minute^c^	7 (30.4)	50 (37.6)	64 (35.4)	23 (52.3)	144 (37.8)
Low Apgar score (<7) at 5 minutes^d^	2 (8.7)	9 (6.8)	18 (10.0)	8 (18.2)	37 (9.7)
Birth weight (kg)^e^					
<1.0	2 (8.3)	3 (2.1)	13 (5.9)	2 (3.9)	20 (4.6)
1.0–2.4	3 (12.5)	24 (16.6)	92 (42.0)	26 (50.0)	145 (33.0)
≥2.5	19 (79.2)	118 (81.4)	114 (52.1)	24 (46.2)	275 (62.5)
NICU admission^f^	8 (34.8)	23 (17.2)	53 (29.6)	17 (34.0)	101 (26.2)
Stillbirth	0 (0.0)	12 (8.2)	31 (14.2)	8 (13.6)	51 (11.4)
Abortions/Perinatal mortality^g^	3 (12.5)	16 (11.0)	48 (21.9)	17 (28.8)	84 (18.8)

EGA, estimated gestational age; NICU, neonatal intensive care unit

^a^Only first twins/triplets were included in cases of multifetal gestation

^b^9 missing values for EGA at delivery

^c^Only live births, 11 missing values for Apgar score at 1 minute

^d^Only live births, 12 missing values for Apgar score at 5 minutes

^e^11 missing values for birth weight

^f^3 missing values for NICU admission, excludes 62 stillbirths

^g^3 missing values for perinatal mortality (2 cases of maternal mortality and 1 loss to follow up)

There were 10 (2.2%) maternal deaths. These cases had received ANC in public health facilities including KATH and were at increased risks if they received less than 4 ANC visits (7.1% vs. 1.6%; p = 0.03). Most (8 out of 10) were referrals from other public health facilities to KATH and had either eclampsia or pre-eclampsia. The immediate clinical causes of the maternal deaths are shown in Table 3. The most common causes were; renal failure, eclampsia and multi-organ failure.

**Table 3:** Final contributory causes of maternal death.

Final Contributory Cause of death	Number (%) (n = 10)
Acute kidney injury	2 (20.0)
Chronic kidney disease	1 (10.0)
Eclampsia	2 (20.0)
Multiple organ failure	2 (20.0)
HELLP syndrome	1 (10.0)
Disseminated intravascular coagulation	1 (10.0)
Cardiac failure	1 (10.0)

HELLP, hemolysis, elevated liver enzymes, low platelets

### Comparison of adverse maternal and perinatal outcomes

Risk estimates for adverse maternal and perinatal outcomes among women with pre-eclampsia/eclampsia compared with women with chronic/gestational hypertension are shown in Table 4. In the final multivariable analysis, women with pre-eclampsia/eclampsia were at significantly higher risks of caesarean section (57.0% vs. 42.4%; adjusted RR, 1.37; 95% CI, 1.01–1.87; p = 0.04), preterm delivery at <34 weeks’ gestation (21.3% vs. 6.5%; adjusted RR, 2.74; 95% CI, 1.40–5.36; p = 0.003) as well as preterm delivery at <37 weeks’ gestation (40.7% vs. 21.3%; adjusted RR, 1.89; 95% CI, 1.25–2.85; p = 0.002), compared to women with chronic/gestational hypertension. Although, the risks of prolonged maternal hospital stay (43.9% vs. 27.1%), NICU admission (30.7% vs. 19.8%) and perinatal mortality (23.5% vs. 11.2%) were significantly higher among women with pre-eclampsia/eclampsia in the univariable analysis, the increased risks did not reach statistical significance after adjusting for confounding.

**Table 4:** Adverse maternal and fetal outcomes of women with pre-eclampsia/eclampsia compared to women with chronic/gestational hypertension.

Maternal and fetal outcomes	Chronic/ Gestational Hypertension N = 170 n (%)	Pre-eclampsia/ Eclampsia N = 281 n (%)	Crude RR (95% CI)	P-value	Adjusted RR (95% CI)	P-value
*Maternal outcomes*						
Caesarean section^a^	72 (42.4)	159 (57.0)	1.41 (1.06, 1.89)	0.02	1.37 (1.01, 1.87)	0.04
Prolonged maternal hospital stay^b^	46 (27.1)	123 (43.9)	1.63 (1.15, 2.28)	0.005	1.40 (0.97, 2.03)	0.07
*Fetal outcomes*						
Preterm delivery						
<34 weeks^c^	11 (6.5)	58 (21.3)	3.26 (1.71, 6.22)	<0.001	2.74 (1.40, 5.36)	0.003
<37 weeks^d^	36 (21.3)	111 (40.7)	1.91 (1.31, 2.78)	0.001	1.89 (1.25, 2.85)	0.002
Low Apgar score (<7) at 1 minute^e^	57 (36.5)	87 (38.7)	1.06 (0.76, 1.48)	0.74	1.16 (0.82, 1.63)	0.40
Low Apgar score (<7) at 5 minutes^f^	11 (7.1)	26 (11.6)	1.65 (0.81, 3.33)	0.17	1.66 (0.81, 3.43)	0.17
NICU admission^g^	31 (19.8)	70 (30.7)	1.55 (1.02, 2.37)	0.04	1.11 (0.72, 1.71)	0.64
Abortions/Perinatal mortality^h^	19 (11.2)	65 (23.5)	2.09 (1.25, 3.48)	0.01	1.07 (0.40, 2.80)	0.90

RR, relative risk; CI, confidence interval; NICU, Neonatal Intensive Care Unit

^a^Adjusted for stage of pregnancy at admission- risk estimates obtained by exclusion of 2 previous caesarean sections, previous extensive myomectomy and major placenta praevia

^b^Adjusted for estimated gestational age (EGA) at diagnosis, EGA at delivery and stage of pregnancy at admission

^c^Adjusted for EGA at diagnosis, number of antenatal visits, and stage of pregnancy at admission

^d^Adjusted for parity (prior to delivery), EGA at diagnosis; number of antenatal visits, and stage of pregnancy at admission

^e^Adjusted for mode of delivery

^f^Adjusted for marital status and mode of delivery

^g^Adjusted for EGA at delivery, mode of delivery and Apgar score at 5 minutes

^h^Adjusted for EGA at delivery and Apgar score at 5 minutes.

## Discussion

This study determined adverse maternal and perinatal outcomes among women with HDPs and directly compared these adverse pregnancy outcomes between women with chronic/gestational hypertension and those with pre-eclampsia/eclampsia. The sociodemographic and reproductive characteristics did not differ significantly between the two groups except for primipaternity, type of facility where most ANC was received, number of ANC visits before, and stage of pregnancy at admission. Women with pre-eclampsia/eclampsia were at increased risk of caesarean section and preterm delivery.

In keeping with the primipaternity concept[16], women with pre-eclampsia/eclampsia were more likely to be carrying their first pregnancy with their index partner compared to those with chronic/gestational hypertension. Women with pre-eclampsia/eclampsia were likely to have had less than 4 ANC visits suggesting that these women were at increased risk of adverse maternal and perinatal outcomes[1, 17]. It is quite reassuring that most women with pre-eclampsia/eclampsia were diagnosed after 27 weeks since the risk of complications and adverse maternal and fetal outcomes increases with decreasing gestational age at diagnosis and delivery[16]. There were no significant differences between the two groups in terms of age (including the extremes of age), parity, multiple pregnancy and obesity.

A study conducted by Crentsil[15] in the same centre about three years earlier found the following percentages of HDPs; chronic hypertension (1.2%), gestational hypertension (22.1%), pre-eclampsia (54.6%) and eclampsia (22.1%). The proportions of pre-eclampsia in her study and our study were quite similar. However, the proportion of chronic/gestational hypertension was higher in the current study, mainly due to increases in the percentages of both chronic and gestational hypertension. Another study in Accra even reported a higher prevalence of chronic/gestational hypertension[18]. This is consistent with the rising incidence of hypertension in the general population in Ghana[19]. It is also possible that more women with HDPs progress to the state of chronic hypertension after delivery. Compared to Crentsil’s study, a smaller proportion of women had eclampsia in our study. This could be an indication that women with hypertension in pregnancy or pre-eclampsia are reporting, being referred or treated/delivered early enough before they progress to eclampsia[1].

Since majority of the women received most of their ANC in other facilities besides KATH and were either referred or reported to KATH on their own, it is conceivable that the diagnosis of HDPs is improving in the peripheral facilities. On the other hand, these facilities and/or their staff may not be fully capable of managing these conditions or the patients were not very confident that these conditions could be adequately managed in the peripheral facilities. It is imperative that the management of HDPs needs to be decentralised to lower level facilities including district and private hospitals, clinics, health centres and maternity homes. Effective decentralisation requires collaborative efforts of KATH and the Ghana Health Service.

The 2.2% maternal mortality in the current study is lower than the 4.1% case fatality rate[4] observed earlier in the same facility three years earlier. Another tertiary facility in Ghana reported a decrease in the proportion of maternal deaths among women with HDPs from 3.9% in 2011[20] to <1% in 2013[5]. These could be indicative of decreasing maternal mortality among women with HDPs in these tertiary facilities in the country. Notwithstanding the decreasing trend, it is worth pointing out that our maternal mortality is still very high compared to similar centres in Ghana and South Africa that have reported maternal mortality rates of about 1%[5, 21]. There is evidence that the management of women with HDPs remains suboptimal in most health facilities in Ghana[5, 22]. Frequent stockouts and lack of essential antihypertensives and anticonvulsants for managing HDP crisis are not uncommon, and this is often aggravated by inadequate laboratory support in most tertiary and other hospitals in Ghana[5, 22], as was the case in this study. The finding that all the cases of maternal mortality were in women with pre-eclampsia/eclampsia is supported by that of another study in Accra where all the maternal deaths attributable to HDPs occurred in women with pre-eclampsia[5]. Therefore, it is imperative that the quality of care provided to women with severe morbidity including HDPs needs to be improved[22, 23]. KATH should have enough trained staff and infrastructure including space and equipment to cope with the high rates of caesarean section and prolonged maternal hospital stay associated with HDPs. To optimize care and outcome for the relatively high proportion of preterm births and improve perinatal mortality rate, the NICU should also have adequate and well-equipped facilities and trained staff, features which are often lacking in low resource settings[2, 24]. For example, although Ray *et al*.[10] had much higher preterm delivery and NICU admission rates in Canada compared to the current study, their perinatal mortality was much lower.

Consistent with the results of previous studies[5, 8, 9, 25], women with pre-eclampsia/eclampsia were at increased risk of caesarean section and preterm delivery at less than 34 weeks’ and 37 weeks’ gestation. The association between type of HDP and prolonged maternal hospital stay was of borderline significance (p = 0.07). In a couple of studies[9, 18], newborns of women with pre-eclampsia/eclampsia were at increased risk of low Apgar scores compared to those of women with chronic/gestational hypertension. Comparatively, a relatively higher proportion of women with chronic/gestational hypertension in the current study had newborns with low Apgar scores at 1 and 5 minutes driving our results in favour of the null hypothesis of no difference. Contrary to the findings of an earlier study in Canada[10], newborns of women with pre-eclampsia/eclampsia were not at increased risk of NICU admission in the current study. This difference is mainly due to a relatively much higher proportion of preterm deliveries and NICU admission rates in the Canada study and may well reflect the different policies related to the timing of delivery for women with pre-eclampsia and NICU admission in a highly resourced country and our low resource setting.

This study had a number of limitations. First, being a tertiary referral hospital, findings from this study cannot be generalised to the rest of the population due to selection bias. Second, women without antenatal records including non-attendees (unbooked women) were excluded since important data pertaining to antenatal care were not available. It is possible that the outcomes and factors associated with these outcomes were different from those of antenatal clinic attendees. However, only 8 women did not have their ANC records or had not booked for ANC, and this might not have changed the overall result significantly. Third, the relatively small number of maternal deaths, made it impossible to quantify the risks associated with this adverse outcome.

## Conclusion

The incidence of adverse maternal and perinatal outcomes among women with HDPs was high. Women with pre-eclampsia/eclampsia were at increased risks of caesarean section and preterm delivery compared to those with chronic/gestational hypertension. Majority of the cases were referrals, emphasizing the need to decentralise the management of these disorders to lower level facilities. Strategies for improving pregnancy outcomes among women with HDPs especially pre-eclampsia/eclampsia in KATH should be explored while the high rates of HDP referrals to KATH are investigated.

## Supporting information

S1 File Dataset. (DTA)

S1 Appendix Questionnaire. (DOC)

## Abbreviations

ANC: antenatal care
CI: confidence interval
EGA: estimated gestational age
HDPs: hypertensive disorders in pregnancy
KATH: Komfo Anokye Teaching Hospital
NICU: neonatal intensive care unit
RR: relative risk
USA: United States of America
