# Supplementary material for: Maternal and perinatal outcomes among women with hypertensive disorders in pregnancy in Kumasi, Ghana
Source: PLoS One. 2019 Oct 4;14(10):e0223478. doi: 10.1371/journal.pone.0223478 (PMC6777792; doi:10.1371/journal.pone.0223478)
Supplement: S1 Appendix — (DOC) [file pone.0223478.s002.doc]

**HDP-QUESTIONNAIRE**

|  |  | Study ID: |  |  |  |  |  |
| --- | --- | --- | --- | --- | --- | --- | --- |

|  | Interview date: | d | d | m | m | y | y |  |
| --- | --- | --- | --- | --- | --- | --- | --- | --- |

**Background data**

| 1. In-patient (IP) number |  |  |  |  |  |  |  |
| --- | --- | --- | --- | --- | --- | --- | --- |

| 1. How old are you now (in completed years)? |  |  |  |  |
| --- | --- | --- | --- | --- |

| 1. What is your marital status now? | | |  |
| --- | --- | --- | --- |
|  | 1. Single | 2. Married/cohabiting |  |
|  | 3. Divorced/separated/widowed | 4. Unknown/not stated |  |

| 1. What is your religion? | | |  |
| --- | --- | --- | --- |
|  | 1. Christian | 2. Moslem |  |
|  | 3. Other (specify).................................................................. | |  |

| 1. What is the highest level of school you have attended? | | | |  |
| --- | --- | --- | --- | --- |
|  | 1. No formal education | 2. Primary/Makaranta | 3. Middle/JSS |  |
|  | 4. Secondary/SSS | 5. Post secondary/higher | ///////////////////////////////// |  |

| 1. What do you do for a living (occupation)? | | |  |
| --- | --- | --- | --- |
|  | 1. Professional-teacher, nurse, accounts, admin | 2. Clerical/secretarial |  |
|  | 3. Vocational-seamstress, hairdresser | 4. Trader/business woman/food seller |  |
|  | 5. Farmer/labourer/domestic worker | 6. Unemployed |  |
|  | 6. Other (specify)................................................................................................. | |  |

| 1. What is your height in cm? (Check from the ANC record or measure) |  |  |  |  |
| --- | --- | --- | --- | --- |

| 1. Check the weight (in kg) at booking from the ANC record |  |  |  | **.** |  |  |
| --- | --- | --- | --- | --- | --- | --- |

| 1. Check and record the haemoglobin (HB), g/dl at the following | | | | | | | |  |
| --- | --- | --- | --- | --- | --- | --- | --- | --- |
|  | Booking HB |  |  | **.** |  |  | 99. Not done |  |
|  | Final HB |  |  | **.** |  |  | 99. Not done |  |
|  | EGA at final HB | w | w | **.** | d |  | 99. Not done |  |

| 1. Please record the dates & times (24hrs/GMT) of admission/readmission from the client folder | | | | | | | |  |
| --- | --- | --- | --- | --- | --- | --- | --- | --- |
|  | Date of admission | d | d | m | m | y | y |  |
|  | Time of admission |  |  | h | h | m | m |  |
|  | Date of readmission | d | d | m | m | y | y |  |
|  | Time of readmission |  |  | h | h | m | m |  |

| 1. Check and record the type of facility the client was referred from/or attended ANC most (if not referred) | | |  |
| --- | --- | --- | --- |
|  | 1.From a public health facility | 2. Private hospital/clinic |  |
|  | 3. Maternity home | 4. KATH client |  |
|  | 5. Other 1 (specify)........................................................................................... | |  |
|  | 6. Other 2 (specify)........................................................................................... | |  |

| 1. If the case was referral from a facility, which facility was she referred from? State name & location, approximate distance (km) and travel time | | | |  |
| --- | --- | --- | --- | --- |
|  | Name & location of referral facility ............................................................................................................................. | | |  |
|  | | Distance | 1. Within Kumasi/ less than 25km |  |
|  | |  | 2. 25-49 km from Kumasi |  |
|  | |  | 3. 50-100km from Kumasi |  |
|  | |  | 4. More than 100km from Kumasi |  |
|  | | Travel time | 1. Less than 30 minutes |  |
|  | |  | 2. 30-60 minutes |  |
|  | |  | 3. 1-2hrs |  |
|  | |  | 4. More than 2 hours |  |

| 1. How many times (including the current pregnancy) have you been pregnant? | | | |  |
| --- | --- | --- | --- | --- |
|  |  |  |  |  |

| 1. What were the outcome(s) of your previous pregnancies? State the number for each outcome | | | |  |
| --- | --- | --- | --- | --- |
|  | Parity (pregnancies carried beyond 28 weeks) |  |  |  |
|  | Preterm deliveries |  |  |  |
|  | Previous caesarean sections |  |  |  |
|  | Spontaneous abortions |  |  |  |
|  | Termination of pregnancies |  |  |  |
|  | Ectopic pregnancies |  |  |  |
| *Write “99” (N/A) if this is her first pregnancy or there was no such outcome* | |  |  |  |

| 1. What was the interval between your last pregnancy/delivery and your current pregnancy (in completed months)? | | | | |  |
| --- | --- | --- | --- | --- | --- |
| *(Skip if this is her first pregnancy)* |  |  |  |  |  |

| 1. Have you ever had high blood pressure (BP) or convulsion in any of your previous pregnancies? | | | | |  |
| --- | --- | --- | --- | --- | --- |
| *(N/A if this is her first pregnancy)* | 1. Yes | 2. No | 88. DK | 99. N/A |  |
| *Skip Q17 (to Q18) if the answer to this question is “No”, “DK” or “N/A”* | | | | |  |

| 1. If you have ever had high blood pressure (BP) or convulsion in any of your previous pregnancies, which disorder was it? | | | |  |
| --- | --- | --- | --- | --- |
|  | High BP or pre-eclampsia | 1. Yes | 2. No |  |
|  | Convulsion (eclampsia) | 1. Yes | 2. No |  |
| *Skip if the answer to Q16 is “No”, “DK” or “N/A”* | | | |  |

| 1. Has your **mother** or any of your **sisters** ever had high blood pressure (BP) or convulsion in any of their pregnancies? | | | |  |
| --- | --- | --- | --- | --- |
| *“No” if she has no sister or sisters have not been pregnant before* | 1. Yes | 2. No | 88. DK |  |

| 1. Is this your first pregnancy with your current partner? | 1. Yes | 2. No |  |
| --- | --- | --- | --- |

| 1. Did you receive donor eggs (oocytes) or sperm for this pregnancy? | | | |  |
| --- | --- | --- | --- | --- |
|  | Donor egg (oocyte) | 1. Yes | 2. No |  |
|  | Donor sperm | 1. Yes | 2. No |  |

| 1. Check and record the number of foetuses in current pregnancy |  |  |  |
| --- | --- | --- | --- |

| 1. Check the number of antenatal clinic visits from the ANC record |  |  |  |
| --- | --- | --- | --- |

| 1. Check and record the estimated gestational age (EGA) at | | | | | | | |  |
| --- | --- | --- | --- | --- | --- | --- | --- | --- |
|  | Booking | 99. N/A |  | w | w | + | d |  |
|  | Diagnosis | 99. N/A |  | w | w | + | d |  |
|  | Admission | 99. N/A |  | w | w | + | d |  |
| *N/A-no ANC record/unknown/delivered before admission* | | | | | |  |  |  |

| 1. State the blood pressure (BP mmHg) at/on | | | | | | |  |
| --- | --- | --- | --- | --- | --- | --- | --- |
|  | Booking | systolic |  |  |  |  |  |
|  |  | diastolic |  |  |  |  |  |
|  | Diagnosis | systolic |  |  |  |  |  |
|  |  | diastolic |  |  |  |  |  |
|  | Admission | systolic |  |  |  |  |  |
|  |  | diastolic |  |  |  |  |  |

| 1. State the stage of pregnancy at admission | | | |  |
| --- | --- | --- | --- | --- |
|  | 1. Antepartum | 2. Intrapartum | 3. Postpartum |  |

| 1. Check and state the hypertensive disorder (HDP) on admission | | |  |
| --- | --- | --- | --- |
|  | 1. Chronic hypertension (HPT) | 2. Gestational hypertension |  |
|  | 3. Pre-eclampsia | 4. Preeclampsia superimposed on chronic HPT |  |
|  | 5. Eclampsia | 6. Postpartum eclampsia |  |

| 1. If client had eclampsia before/after admission, state the date & time (24hrs/GMT) of first convulsion | | | | | | | |  |
| --- | --- | --- | --- | --- | --- | --- | --- | --- |
|  | Date | d | d | m | m | y | y |  |
|  | Time |  |  | h | h | m | m |  |

| 1. If client had eclampsia, state the number of fits before and after the administration of MgS0_4_ | | | | | |  |
| --- | --- | --- | --- | --- | --- | --- |
|  | Before MgS0_4_ |  |  |  |  |  |
|  | After MgS0_4_ |  |  |  |  |  |

| 1. If the client was referred as a case of severe pre-eclampsia or eclampsia, state the number of doses of MgS0_4_ she was given before referral | | |  |
| --- | --- | --- | --- |
| *If MgS0_4_  was not given then write “0”* |  |  |  |

| 1. Check and record whether the client had the following clinical symptoms & signs or complications | | | | |  |
| --- | --- | --- | --- | --- | --- |
|  | Headache | 1. Yes | 2. No | 99. N/A |  |
|  | Visual disturbances | 1. Yes | 2. No | 99. N/A |  |
|  | Epigastric/RUQ pain/tenderness | 1. Yes | 2. No | 99. N/A |  |
|  | Pulmonary edema | 1. Yes | 2. No | ///////////// |  |
|  | Pulmonary embolism | 1. Yes | 2. No | ///////////// |  |
|  | HELLP syndrome | 1. Yes | 2. No | ///////////// |  |
|  | DIC | 1. Yes | 2. No | ///////////// |  |
|  | Renal failure | 1. Yes | 2. No | ///////////// |  |
|  | Intracranial hemorrhage/stroke | 1. Yes | 2. No | ///////////// |  |
|  | Other (specify)..................................................................................... | | | |  |
| *N/A (not applicable) for unconscious patients* | | | | | |

| 1. Which of the following medical conditions did the client have on admission? Check from the client notes | | | |  |
| --- | --- | --- | --- | --- |
|  | Diabetes Mellitus | 1. Yes | 2. No |  |
|  | Respiratory diseases/asthma | 1. Yes | 2. No |  |
|  | Cardiac conditions | 1. Yes | 2. No |  |
|  | Anaemia *(not from acute hemorrhage)* | 1. Yes | 2. No |  |
|  | Shock | 1. Yes | 2. No |  |
|  | Fever (confirm on temp chart) | 1. Yes | 2. No |  |
|  | Pyelonephritis | 1. Yes | 2. No |  |
|  | Sepsis/parenteral antibiotics given | 1. Yes | 2. No |  |
|  | Other (specify).................................................................... | | |  |

| 1. Did the client have the following conditions? Check from the client notes | | | |  |
| --- | --- | --- | --- | --- |
|  | Premature rupture of membranes (PROM) | 1. Yes | 2. No |  |
|  | Oligohydramnios (AFI<5cm) | 1. Yes | 2. No |  |
|  |  |  |  |  |

| 1. First laboratory/bedside investigations done on admission | | | | | | | | | | |  |
| --- | --- | --- | --- | --- | --- | --- | --- | --- | --- | --- | --- |
|  | Urine protein | Number of (+) | 00. N/A |  |  |  |  |  | + |  | |
|  | Haematology | HB (g/dl) | 00. N/A |  |  |  |  | **.** |  |  | |
|  |  | Platelets (x10^9^/L) | 00. N/A |  |  |  |  |  |  |  | |
|  |  | Clotting time (mins) | 00. N/A |  |  |  |  |  |  |  | |
|  | Uric acid | (SI) (µmol/L) | 00. N/A |  |  |  |  | **.** |  |  | |
|  | RFTs | BUN (SI) (mmol/L) | 00. N/A |  |  |  |  | **.** |  |  | |
|  |  | Creat (SI) (µmol/L) | 00. N/A |  |  |  |  | **.** |  |  | |
|  | LFTs | GOT (AST) (U/L) | 00. N/A |  |  |  |  | **.** |  |  | |
|  |  | GPT (ALT) (U/L) | 00. N/A |  |  |  |  | **.** |  |  | |
|  |  | Alkaline phos (U/L) | 00. N/A |  |  |  |  | **.** |  |  | |
|  |  | LDH (U/L) | 00. N/A |  |  |  |  |  |  |  | |
|  |  | GGT (U/L) | 00. N/A |  |  |  |  | **.** |  |  | |
|  |  | Bili-total (SI) (µmol/L) | 00. N/A |  |  |  |  | **.** |  |  | |
|  |  | Bili-direct (SI) (µmol/L) | 00. N/A |  |  |  |  | **.** |  |  | |
|  |  | Protein-total (SI) (g/L) | 00. N/A |  |  |  |  | **.** |  |  | |
|  |  | Albumin (SI) (g/L) | 00. N/A |  |  |  |  | **.** |  |  | |
|  |  | Globulin (g/dL) | 00. N/A |  |  |  |  | **.** |  |  | |
|  |  | Bili-indirect (SI) (µmol/L) | 00. N/A |  |  |  |  | **.** |  |  | |

| 1. Management/treatment given | | | | |  |
| --- | --- | --- | --- | --- | --- |
|  | Anticonvulsants (MgS0_4_) | 1. Yes | 2. No | 99. N/A |  |
|  | Antihypertensives | 1. Yes | 2. No | 99. N/A |  |
|  | Corticosteroids (Dexamethasone) | 1. Yes | 2. No | 99. N/A |  |
|  | Antibiotics | 1. Yes | 2. No | 99. N/A |  |
|  | Induction of labour (IOL) | 1. Yes | 2. No | 99. N/A |  |
|  | Augmentation of labour | 1. Yes | 2. No | 99. N/A |  |
|  | Caesarean section (CS)/hysterotomy | 1. Yes | 2. No | 99. N/A |  |
|  | Hysterectomy | 1. Yes | 2. No | 99. N/A |  |
|  | Haemotransfusion | 1. Yes | 2. No | 99. N/A |  |
|  | Haemodialysis | 1. Yes | 2. No | 99. N/A |  |
|  | Intravenous fluids (IVFs) | 1. Yes | 2. No | 99. N/A |  |
|  | Central venous line | 1. Yes | 2. No | 99. N/A |  |

| 1. If antihypertensives were given, which antihypertensives were given? | | | |  |
| --- | --- | --- | --- | --- |
|  | Nifedipine (Tabs) | 1. Yes | 2. No |  |
|  | Nifedipine (Sublingual) | 1. Yes | 2. No |  |
|  | Methyldopa (Aldomet) | 1. Yes | 2. No |  |
|  | Hydralazine (IV) | 1. Yes | 2. No |  |
|  | Labetalol | 1. Yes | 2. No |  |

| 1. If haemotransfused, state the number of units haemotransfused | | | |  |
| --- | --- | --- | --- | --- |
|  | Blood |  |  |  |
|  | Fresh frozen plasma (FFP) |  |  |  |

| 1. If IV fluids were given, state number of **mls** of fluids given | | | | | | |  |
| --- | --- | --- | --- | --- | --- | --- | --- |
|  | Crystalloids (NS, DS, RL) |  |  |  |  |  |  |
|  | Colloids (haemacele etc) |  |  |  |  |  |  |

| 1. If induction of labour was done at KATH, state the date & time (24hrs/GMT) | | | | | | | |  |
| --- | --- | --- | --- | --- | --- | --- | --- | --- |
|  | Date of IOL | d | d | m | m | y | y |  |
|  | Time of IOL |  |  | h | h | m | m |  |

| 1. Check and write the dates & time (24hrs/GMT) of vaginal delivery at KATH | | | | | | | |  |
| --- | --- | --- | --- | --- | --- | --- | --- | --- |
|  | Date | d | d | m | m | y | y |  |
|  | Time (2^nd^ stage for vag delivery) |  |  | h | h | m | m |  |
| *If delivered outside KATH & time of delivery is not known, leave “TIMEDEL” blank* | | | | | | |  |  |

| 1. Check & record the duration of labour in minutes (for vaginal deliveries & emergency CS) at KATH | | | | | | | |  |
| --- | --- | --- | --- | --- | --- | --- | --- | --- |
|  | First stage |  |  |  |  |  |  |  |
|  | Second stage |  |  |  |  |  |  |  |
| *Not applicable in emergency CS* | Third stage |  |  |  |  |  |  |  |
|  | Total duration |  |  |  |  |  |  |  |
| *If emergency CS, first stage is till decision time* | | | |  |  |  |  |  |

| 1. State the estimated gestational age (EGA) in weeks at the following | | | | | | | |  |
| --- | --- | --- | --- | --- | --- | --- | --- | --- |
|  | Delivery | 99. N/A |  | w | w | + | d |  |
|  | Discharge | 99. N/A |  | w | w | + | d |  |
|  | Death | 99. N/A |  | w | w | + | d |  |

| 1. Check and write the mode of delivery | | |  |
| --- | --- | --- | --- |
|  | 1. Spont vertex delivery (SVD) | 2. Assisted vacuum delivery |  |
|  | 3. Elective CS/hysterotomy | 4. Emergency CS/hysterotomy |  |

| 1. What was/were the birth weight(s) in grammes (g) | | | | | | | |  |
| --- | --- | --- | --- | --- | --- | --- | --- | --- |
|  | Baby 1 |  |  |  |  |  |  |  |
|  | Baby 2 |  |  |  |  |  |  |  |
|  | Baby 3 |  |  |  |  |  |  |  |

| 1. What was/were the outcome(s) of delivery | | | |  |
| --- | --- | --- | --- | --- |
|  | Baby 1 | 1. Live birth | 2. Still birth (SB) |  |
|  | Baby 2 | 1. Live birth | 2. Still birth (SB) |  |
|  | Baby 3 | 1. Live birth | 2. Still birth (SB) |  |

| 1. What was/were the sex of the babies? | | | |  |
| --- | --- | --- | --- | --- |
|  | Baby 1 | 1. Male | 2. Female |  |
|  | Baby 2 | 1. Male | 2. Female |  |
|  | Baby 3 | 1. Male | 2. Female |  |

| 1. What were the Apgar scores at the first & fifth mins? | | | | | |  |
| --- | --- | --- | --- | --- | --- | --- |
|  | | Apgar score at 1min |  |  |  |  |
|  | | Apgar score at 5min |  |  |  |  |
|  | Twin 2 | Apgar score at 1min |  |  |  |  |
|  | Twin 2 | Apgar score at 5min |  |  |  |  |
| *If singleton, write “NA” for twin2 Apgar scores, if baby was still born record Apgar as “0”* | | | | |  |  |

| 1. If the baby was still born, when did the foetus die? | | |  |
| --- | --- | --- | --- |
|  | 1. Before labour (IUFD) | 2. During labour |  |

| 1. What was the estimated blood loss (in mls) at delivery? |  |  |  |  |  |
| --- | --- | --- | --- | --- | --- |

| 1. Did the client have antepartum or postpartum hemorrhage (APH or PPH)? | | | |  |
| --- | --- | --- | --- | --- |
|  | Antepartum hemorrhage (APH) | 1. Yes | 2. No |  |
|  | Postpartum hemorrhage (PPH) | 1. Yes | 2. No |  |

| 1. Please record the dates & times (24hrs/GMT) of discharge /death of the mother | | | | | | | |  |
| --- | --- | --- | --- | --- | --- | --- | --- | --- |
|  | Date of discharge | d | d | m | m | y | y |  |
|  | Date of discharge (readmission) | d | d | m | m | y | y |  |
|  | Date of death | d | d | m | m | y | y |  |
|  | Time of death |  |  | h | h | m | m |  |

| 1. If the mother died, state the cause of death (if known) | |  |
| --- | --- | --- |
| 1. |  |  |
| 2. |  |  |
| 3. |  |  |

| 1. Please record the dates of the first and final postnatal reviews for mothers who were discharged | | | | | | | |  |
| --- | --- | --- | --- | --- | --- | --- | --- | --- |
|  | Date of first review | d | d | m | m | y | y |  |
|  | Date of final review | d | d | m | m | y | y |  |

| 1. Please record the following at the final postnatal review of the mother | | | | | | |  |
| --- | --- | --- | --- | --- | --- | --- | --- |
|  | Final review systolic BP (mmHg) |  | |  |  |  |  |
|  | Final review systolic BP (mmHg) |  | |  |  |  |  |
|  | Was the client still on antihypertensives? |  | 1. Yes | | 2. No | |  |

| 1. Was the baby admitted to the Neonatal Intensive Care Unit (NICU)? | | |  |
| --- | --- | --- | --- |
|  | 1. Yes | 2. No |  |
